# Inpatient Burden, Costs, and Characteristics of Peripheral Artery Disease in Slovenia

**DOI:** 10.2478/sjph-2026-0017

**Published:** 2026-09-01

**Authors:** Tjaša Furlan, Borut Jug, Jerneja Farkaš-Lainščak, Dalibor Gavrić, Irena Ograjenšek, Petra Došenović Bonča

**Affiliations:** Faculty of Medicine, University of Ljubljana, Vrazov trg 2, 1000 Ljubljana, Slovenia; General Hospital Trbovlje, Rudarska cesta 9, 1420 Trbovlje, Slovenia; Department of Vascular Diseases, University Medical Centre Ljubljana, Zaloška cesta 7, 1000 Ljubljana, Slovenia; General Hospital Murska Sobota, Ulica dr. Vrbnjaka 6, Rakičan, 9000 Murska Sobota, Slovenia; National Institute of Public Health, Trubarjeva cesta 2, 1000 Ljubljana, Slovenia; Health Insurance Institute of Slovenia, Miklošičeva cesta 24, 1000 Ljubljana, Slovenia; School of Economics and Business, University of Ljubljana, Kardeljeva ploščad 17, 1000 Ljubljana, Slovenia

**Keywords:** Peripheral artery disease, Chronic limb-threatening ischaemia, Intermittent claudication, Hospitalization, Mortality, periferna arterijska bolezen, kronična kritična ishemija, intermitentna klavdikacija, hospitalizacija, umrljivost

## Abstract

**Introduction:**

We assessed inpatient burden, costs, demographics, comorbidity patterns, and outcomes of peripheral artery disease (PAD) in Slovenia.

**Methods:**

We included patients hospitalised for PAD (primary diagnosis, International Classification of Diseases, 10th revision, code I70.2–I70.9) in Slovenia between January 1st, 2015, and December 31st, 2022. We estimated crude and age-standardised hospitalisation rates, hospitalisation reimbursement costs, patient characteristics (age and sex distribution, disease severity and prevalence of comorbidities), uptake of revascularisation, major amputations and in-hospital mortality.

**Results:**

We included 15,978 patients (60% men, median age 72 years, 32% chronic limb-threatening ischaemia) with 27,139 hospital episodes, yielding a mean of ~3,400 hospitalisations per year (hospitalisation rate 161 per 100,000/year). Median direct cost (inflation-adjusted to 2025) per hospital episode was €3,177, yielding total costs of €10.8 million/year. The most common comorbidities were arterial hypertension (35%), dyslipidemia (21%) and diabetes mellitus (19%), with a significantly higher prevalence in patients with chronic limb-threatening ischaemia. During the observation period, revascularisation procedures increased from 77.5% to 80.2%, while major amputations decreased from 13.2% to 7.9%, and mortality decreased from 5.0% to 3.3%.

**Conclusions:**

The inpatient burden of PAD is considerable in Slovenia. Patients hospitalised for PAD have a high burden of comorbidities, especially when presenting with chronic limb-threatening ischaemia; nonetheless, in-hospital major amputations and mortality decreased substantially over the last decade.

## Izvleček

**Uvod:**

Ocenili smo stopnjo hospitalizacij, direktne stroške, značilnosti bolnikov in breme sočasnih bolezni ter bolnišnične izide zaradi periferne arterijske bolezni (PAB) v Sloveniji.

**Metode:**

V analizo smo vključili bolnike, hospitalizirane zaradi PAB (glavna diagnoza I70.2–I70.9 po Mednarodni klasifikaciji bolezni, 10. izdaja) v Sloveniji med 1. januarjem 2015 in 31. decembrom 2022. Ocenili smo stopnjo hospitalizacij, direktne stroške, značilnosti bolnikov (starost, spol, izraženost bolezni in sočasne bolezni) ter delež revaskularizacij, velikih amputacij uda in bolnišnično umrljivost.

**Rezultati:**

Vključili smo 15.978 bolnikov (60 % moških, mediana starost 72 let, 32 % s kronično kritično ishemijo uda) s 27.139 hospitalizacijami, kar predstavlja povprečno ~3.400 hospitalizacij/leto (stopnja hospitalizacij 161,4 na 100.000 prebivalcev/leto). Mediani direktni stroški (prilagojeni za inflacijo do 2025) so bili 3.177 evrov za hospitalno epizodo oziroma 10,8 milijona evrov/leto. Najpogostejše sočasne bolezni so bile arterijska hipertenzija (35 %), dislipidemija (21 %) in sladkorna bolezen (19 %); statistično značilno so bile pogostejše pri bolnikih s kronično kritično ishemijo uda. V opazovanem obdobju je delež revaskularizacij narasel s 77,5 % na 80,2 %, medtem ko je delež velikih amputacij upadel s 13,2 % na 7,9 %, umrljivost pa s 5,0 % na 3,3 %.

**Zaključki:**

Breme hospitalizacij zaradi PAB je v Sloveniji precejšnje. Hospitalizirani bolniki s PAB imajo veliko breme sočasnih bolezni, zlasti ko gre za kronično kritično ishemijo uda. Kljub temu so velike amputacije in bolnišnična umrljivost pri bolnikih s PAB v zadnjem desetletju znatno upadle.

## 1 INTRODUCTION

Peripheral artery disease (PAD) is defined as the progressive involvement of large and medium-sized arteries other than those supplying the heart (i.e., coronary artery disease) or the brain (i.e., cerebrovascular disease) (1). Most commonly, PAD refers to atherosclerotic involvement of lower extremity arteries. As a manifestation of atherosclerotic vascular disease, PAD is associated with both a very high risk of acute cardiovascular events unrelated to extremity arteries and a wide range of clinical presentations due to peripheral blood flow obstruction (2, 3). While more than 50% of affected individuals are asymptomatic (i.e., Fontaine stage I), the majority of patients present with symptoms of intermittent claudication (IC, i.e., Fontaine stage II) (4). Chronic limb-threatening ischaemia (CLI) represents a more severe chronic PAD presentation and underlies poor limb outcomes (4) — patients with CLI present with a critical hemodynamic status responsible for ischemic rest pain (Fontaine stage III) or foot gangrene (Fontaine stage IV) (5).

PAD represents a significant public health burden. An estimated 113 million adults aged ≥ 40 years had PAD globally in 2019, with a prevalence of 1.52% (5). The estimated prevalence of PAD varies across populations, definitions, and diagnostic criteria. For instance, the estimated prevalence is higher in older populations (e.g., 14.9% in those aged 80–84 years) (5) and in populations with a higher burden of cardiovascular risk factors, especially those specifically associated with PAD, such as diabetes mellitus, smoking, arterial hypertension, and dyslipidemia (6). The prevalence is ~3 times higher when asymptomatic PAD is taken into account (e.g., screening by reduced ankle-brachial index in community-based cohort studies) and ~10 times higher in the outpatient setting than in the inpatient setting (annual PAD prevalence 11.8% versus 1.6%) (7) following coronary artery disease and stroke. This study provides the first comparison of the prevalence of peripheral artery disease between high-income countries (HIC.

In Slovenia, current data on the burden of PAD remain very limited. To date, only two cohort studies conducted in selected subpopulations have provided estimates of PAD burden (8, 9), and none at the level of the entire population. To assess the burden of PAD at the level of the entire Slovenian population, and to support service planning as well as to inform clinical and public health strategies for atherosclerotic cardiovascular disease, the objective of our study was to quantify the national inpatient burden of PAD in Slovenia using available routinely collected hospital data.

## 2 METHODS

### 2.1 Study design

The study was designed at two levels—population and individual—both based on routinely collected hospital data. The population-level covered several aspects of PAD burden—dynamics of hospitalisation rates, the share of episodes requiring procedures, the share of episodes ending with an unfavourable outcome, and costs, while the individual-level addressed available PAD risk factors and associated comorbidities.

### 2.2 Data source

Routinely collected electronic hospital discharge data from the National Hospital Health Care Statistics (NHHCS) database were used. Data for the 8-year period from January 1st, 2015, to December 31st, 2022, were captured (10).

The database is maintained by the Health Insurance Institute of Slovenia (single-payer for universal compulsory health insurance). It contains episode-level information required for reimbursement (e.g., demographics, admission/discharge dates, discharge status, principal diagnosis and recorded comorbidities, and coded procedures used for case-mix/diagnosis-related group assignment). Thus, the basic record in NHHCS database is a hospitalisation due to a disease episode.

### 2.3 Case definition of PAD

PAD was defined according to the International Classification of Diseases, Tenth Revision (ICD-10) (11). Codes I70.2–I70.9 were included (Table 1). PAD severity was categorised using the Fontaine stage as recorded in the discharge diagnosis coding (Table 1).

**Table 1:** Peripheral artery disease codes based on the International Classification of Diseases, Tenth Revision.

**Diagnosis**	**ICD-10 code**
Atherosclerosis of extremity arteries	I70.2
- unspecified	I70.20
- with intermittent claudication (Fontaine stage IIa)	I70.21
- with rest pain (Fontaine stage IIb)	I70.22
- with ulceration (Fontaine stage III)	I70.23
- with gangrene (Fontaine stage IV)	I70.24
Atherosclerosis of other arteries	I70.8
Generalised and undefined atherosclerosis	I70.9

Legend: ICD-10 – International Classification of Diseases, Tenth Revision

### 2.4 Population-level methods

#### 2.4.1 Population-level study design, selection of records for analysis and unit of observation

This part of the study was designed as a temporal pattern study. For this study, all hospital inpatient care episodes of individuals > 18 years of age in Slovenia with a primary diagnosis of PAD were extracted from the NHHCS database. The basic records were subsequently aggregated into predefined time units. In most analyses, the time unit of observation was 1 year, while in selected analyses it was six months.

#### 2.4.2 Observed outcomes and consideration of confounding factors

The observed outcomes at the population level were various indicators.

**Crude hospitalisation rates:** Crude hospitalisation rates were calculated as the number of PAD hospital episodes in a given calendar year divided by the mid-year adult population (≥ 18 years) of Slovenia and expressed per 100,000 persons. Episode records were aggregated at the predefined 1-year observation time unit.

**Age-standardised hospitalisation rates:** This is an indicator in which age is considered as a confounder. Age-standardised hospitalisation rates were calculated by direct standardisation using age-specific rates and standard population weights for the age groups 18–49, 50–59, 60–64, 65–79, and ≥ 80 years (population counts from the Statistical Office of the Republic of Slovenia). Episode records were aggregated for this indicator at the predefined half-year observation time unit.

**Share of episodes requiring procedures:** This indicator was defined as the percentage of episodes requiring procedures. Included was any lower-limb revascularisation, categorised as endovascular and/or open surgical based on procedure coding recorded for the hospital episode. Episode records were aggregated at the predefined 1-year observation time unit.

**Share of episodes ended with an unfavourable outcome:** In this context, two indicators were defined: a percentage of episodes terminated with major lower-limb amputation and a percentage of episodes terminated with death during hospitalisation (discharge status). Episode records were aggregated at the predefined 1-year observation time unit.

**Direct reimbursement costs:** Direct reimbursement costs were derived from episode-level payer reimbursement recorded in the database (hospital payment/case-mix-based reimbursement) and represent direct inpatient costs from the payer perspective. Costs are reported and summarised as the median cost per episode and the total cost per year.

#### 2.4.3 Methods of analysis

Time patterns/dynamics were visualised using sequence plots and, based on the graphical display, were visually analysed descriptively.

### 2.5 Individual-level methods

#### 2.5.1 Individual-level study design, definition of point of observation and unit of observation

This part of the study was designed as a cross-sectional study. The first episode in the observed period — the first hospital admission (index admission) — was the identifying point for patient-level analysis. As a result, the individual patient became the unit of observation.

#### 2.5.2 Outcome variable

The individual-level outcome variable was PAD severity, defined as the presence of CLI (binary outcome variable based on Fontaine stages: 1 = stages III and IV; 0 = stages I and II or unspecified PAD).

#### 2.5.3 Risk factors and comorbidities (patients' characteristics)

Two risk factors, being sex (0 = female, 1 = male) and age (years), and thirteen selected comorbidities (Table 2) were included in the study. Binary indicators (0 = no, 1 = yes) were created for all comorbidities.

**Table 2:** Comorbidity codes based on the International Classification of Diseases, Tenth Revision.

**Diagnosis**	**ICD-10 codes**
Coronary artery disease	I20.0 – I25.9
Cerebrovascular disease	I63.0 – I66.9
Aortic aneurysm	I71.0 – I71.9
Diabetes mellitus	E09.21 – E16.9
Arterial hypertension	I10 – I13.9
Dyslipidemia	E78.0 – E78.9
Heart failure	I50.0 – I50.9
Atrial fibrillation	I48
Chronic kidney disease	N10 – N19
Cancer	C00.1 – C96.9; D00.1 – D48.9
COPD/asthma	J44.0 – J46
Dementia	F00.0 – F03; F05.1
Depression	F06.4; F32 – F33.9

Legend: ICD-10 – International Classification of Diseases, Tenth Revision; COPD – chronic obstructive pulmonary disease

Both risk factors and comorbidities were extracted from the NHHCS database at the time of index admission.

#### 2.5.4 Statistical analysis

Baseline characteristics of the study group (patient-level data) were summarised using descriptive statistics. We assessed data for normality distribution using the Kolmogorov-Smirnov test. Descriptive statistics were calculated to summarise demographic and clinical characteristics, using median with interquartile range for age (continuous variable) and frequencies with percentages for categorical variables.

Univariate analysis of the association between the observed outcome and risk factors and comorbidities was performed using logistic regression; results are reported as odds ratios (ORs) with 95% confidence intervals and corresponding p-values.

### 2.6 Analytical tools

Statistical analysis was performed with R, version 4.4.0 (2023, Foundation for Statistical Computing, Vienna, Austria).

### 2.7 Statement of ethics

The study protocol was reviewed and approved by the National Medical Ethics Committee of the Republic of Slovenia, Ministry of Health, Republic of Slovenia (approval number KME-0120-29/2022/6). The study was conducted in accordance with the Declaration of Helsinki. The requirement for informed consent was waived by the National Medical Ethics Committee of the Republic of Slovenia, Ministry of Health, Republic of Slovenia.

## 3 RESULTS

### 3.1 Description of basic data

During the observation period, 27,139 hospitalisations due to PAD episodes were recorded in 15,987 patients in the NHHCS database.

### 3.2 Results of population-level analysis

#### 3.2.1 Crude PAD hospitalisation rates

Annual hospitalisation rates fluctuated between 183.8 per 100,000 inhabitants per year (hereinafter 100,000/year) (age-standardised 113.7 per 100,000/year) in 2019 and 142 per 100,000/year (age-standardised 87.5 per 100,000/year) in 2022 (Table 3).

**Table 3:** Annual PAD hospitalisation rates per 100,000 adult population in Slovenia, 2015–2022.

**Year**	**Total**			**Fontaine I/II/unspecified PAD**		**Fontaine III/IV**	
		
	**N**	**Hosp. rate**	**Age-standardised hosp. rate**	**N**	**Hosp. rate**	**N**	**Hosp. rate**
2015	3,462	167.7	102.8	2,264	109.7	1,198	58.0
2016	3,218	154.8	95.6	2,090	100.2	1,128	54.5
2017	3,459	166.4	102.8	2,285	110.1	1,174	56.8
2018	3,611	173.1	107.3	2,461	118.6	1,150	55.4
2019	3,858	183.8	113.7	2,642	125.5	1,216	58.2
2020	3,102	146.5	90.8	2,076	98.7	1,026	48.8
2021	3,000	142.3	87.5	2,009	95.3	991	47.0
2022	3,429	161.3	100.1	2,284	107.1	1,145	54.2
TOTAL	27,139	162.7	99.7	18,111	108.6	9,028	54.1

#### 3.2.2 Age-standardised PAD hospitalisation rates

**Figure 1:**
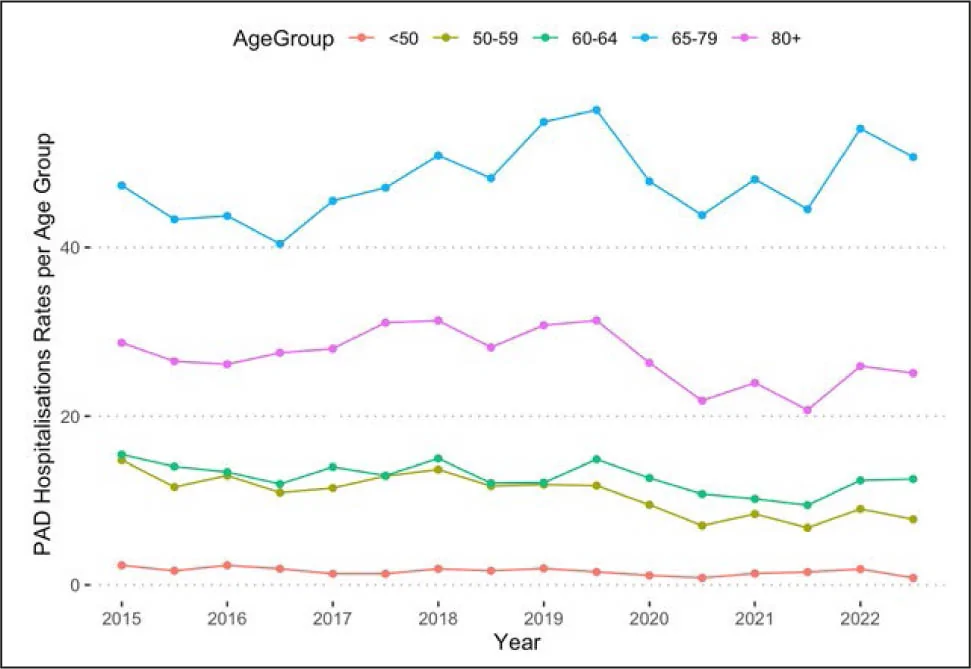
Semiannual (6-month) pattern of peripheral artery disease (PAD) hospitalisation rates per 100,000 population by age group, Slovenia 2015–2022.

The lowest hospitalisation rates were observed in patients aged less than 50 years, while highest hospitalisation rates were in patients aged 65–79 years (Figure 1).

#### 3.2.3 Share of episodes requiring procedures or ended with an unfavourable outcome

Overall, 78.5% of hospital episodes included a revascularisation procedure (52.9% endovascular, 31.9% surgical, and 6.3% both during a single hospital episode). Across the study period, procedure and outcome rates varied year-to-year (Table 4).

**Table 4:** Share of episodes (N = 27,139) requiring procedures or ended with an unfavourable outcome in the study of the burden of inpatient PAD, Slovenia 2015–2022.

**Year**	**Episodes requiring procedures (%)**			**Episodes ended with an unfavourable outcome (%)**	
	
	**Any procedure**	**Endovascular**	**Surgery**	**Amputation**	**Death**
2015	77.5%	48.8%	33.9%	13.2%	5.0%
2016	76.2%	50.5%	32.1%	11.8%	4.6%
2017	75.9%	50.5%	31.6%	10.0%	4.0%
2018	78.0%	57.2%	26.9%	9.2%	3.8%
2019	78.1%	54.1%	30.4%	9.3%	3.6%
2020	79.7%	53.4%	34.0%	9.6%	3.4%
2021	82.7%	55.1%	34.1%	9.7%	3.6%
2022	80.2%	53.6%	32.7%	7.9%	3.3%

Legend: Rate (% of hospital episodes) with revascularisation (any, endovascular, or surgical), major amputation, or in-hospital death

A major amputation occurred in 10.1% of episodes overall (13.2% in 2015 and 7.9% in 2022), and the overall share of episodes ending in death was 3.9% (5.0% in 2015 and 3.3% in 2022). This share was lower in intermittent claudication (Fontaine stages I/II/unspecified) than in chronic limb-threatening ischaemia (Fontaine stages III/IV) (1.1% vs 8.7%).

#### 3.2.4 PAD reimbursement costs

Direct annual reimbursement costs in the period 2015-2022 ranged between a median of EUR 2,401.98 per hospital episode and a median of EUR 3,775.64 per hospital episode, resulting in total direct inpatient reimbursement costs for PAD management of between EUR 13.7 and EUR 17.5 million per year (Table 5).

**Table 5:** Annual cost summary for PAD management, Slovenia 2015–2022.

**Year**	**Episodes (N)**	**Total cost (Eur)**	**Median (Q1–Q3) cost per episode (Eur)**
2015	3,462	15,677,050.92	3389.68 (2116.55–6304.64)
2016	3,218	13,926,149.84	3013.47 (1729.88–6167.16)
2017	3,459	13,739,039.27	2401.98 (1679.99–5603.43)
2018	3,611	14,704,501.30	2544.57 (1781.99–5382.35)
2019	3,858	17,522,197.99	2686.01 (1939.18–5857.11)
2020	3,102	14,440,400.55	3141.08 (1928.60–6287.42)
2021	3,000	14,588,474.86	3775.64 (2001.43–6265.79)
2022	3,429	17,382,561.56	3056.31 (2214.33–7080.68)

Legend: Q1–Q3 – interquartile range

### 3.3 Results of individual-level analysis

#### 3.3.1 Basic description of a group of patients

Out of the 15,987 patients included in the analysis, 62.2% had one hospital episode, 22.6% had two hospital episodes, and 15.2% had three or more hospital episodes. Sixty percent were men, and the median age was 72 years; 10,948 (68.5%) had a primary diagnosis of Fontaine stage I or II (IC) or unspecified PAD, and 5,039 (31.5%) had a primary diagnosis of CLI. See Table 6 and Table 7.

**Table 6:** Characteristics of 15,987 patients included in the study of the burden of inpatient PAD, Slovenia 2015–2022.

**Characteristic**	**Category**	**N (%) / Me (Q1–Q3)**
**SEVERITY OF PAD**		
Fontaine stage	Unspecified	2,301 (14.4%)
	Fontaine IIa	1,125 (7.0%)
	Fontaine IIb	7,522 (47.1%)
	Fontaine III	2,085 (13.0%)
	Fontaine IV	2,954 (18.5%)
**RISK FACTORS**		
Sex	Male	9,600 (60.1%)
Age (years)		72 (64–81)
**COMORBIDITIES**		
Coronary artery disease	Present	1,209 (7.6%)
Cerebrovascular disease	Present	335 (2.1%)
Aortic aneurysm	Present	149 (0.9%)
Diabetes mellitus	Present	3,006 (18.8%)
Arterial hypertension	Present	5,577 (34.9%)
Dyslipidemia	Present	3,294 (20.6%)
Heart failure	Present	867 (5.4%)
Atrial fibrillation	Present	1,356 (8.5%)
Chronic kidney disease	Present	1,372 (8.6%)
Cancer	Present	399 (2.5%)
COPD/asthma	Present	575 (3.6%)
Dementia	Present	396 (2.5%)
Depression	Present	57 (0.4%)

Legend: COPD – chronic obstructive pulmonary disease; Me – median; Q1–Q3 – interquartile range

**Table 7:** Results of univariate logistic regression analysis of the association between presence of CLI and patients' risk factors/comorbidities in 15,987 patients in the study of the burden of inpatient PAD, Slovenia 2015–2022.

**Characteristic**	**Category**	**N_CLI_ / N_cat_ (%) / Me (Q1–Q3)**	**OR**	**95% CI**	**p-value**
**RISK FACTORS**					
Sex	female	2,288/6,387 (35.8%)	1.00		
	male	2,751/9,600 (28.7%)	0.72	0.67, 0.77	< 0.001
Age (years)		79 (69, 85)	1.06	1.06, 1.07	< 0.001
**COMORBIDITIES**					
Coronary artery	no	4,685/14,778 (31.7%)	1.00		
disease	yes	354/1,209 (29.3%)	0.89	0.78, 1.01	0.081
Cerebrovascular	no	4,937/15,652 (31.5%)	1.00		
disease	yes	102/335 (30.4%)	0.95	0.75, 1.20	0.670
Abdominal aortic	no	5,016/15,838 (31.7%)	1.00		
aneurysm	yes	23/149 (15.4%)	0.39	0.25, 0.60	< 0.001
Diabetes mellitus	no	3,700/12,981 (28.5%)	1.00		
	yes	1,339/3,006 (44.5%)	2.01	1.86, 2.19	< 0.001
Arterial	no	3,182/10,410 (30.6%)	1.00		
hypertension	yes	1,857/5,577 (33.3%)	1.13	1.06, 1.22	< 0.001
Hyperlipidemia	no	4,243/12,693 (33.4%)	1.00		
	yes	796/3,294 (24.2%)	0.63	0.58, 0.69	< 0.001
Heart failure	no	4,492/15,120 (29.7%)	1.00		
	yes	547/867 (63.1%)	4.04	3.51, 4.67	< 0.001
Atrial fibrillation	no	4,355/14,631 (29.8%)	1.00		
	yes	684/1,356 (50.4%)	2.40	2.15, 2.69	< 0.001
Chronic kidney	no	4,319/14,615 (29.6%)	1.00		
disease	yes	720/1,372 (52.5%)	2.63	2.35, 2.94	< 0.001
Cancer	no	4,847/15,588 (31.1%)	1.00		
	yes	192/399 (48.1%)	2.06	1.68, 2.51	< 0.001
COPD	no	4,872/15,412 (31.6%)	1.00		
	yes	167/575 (29.0%)	0.89	0.74, 1.06	0.193
Dementia	no	4,745/15,591 (30.4%)	1.00		
	yes	294/396 (74.2%)	6.59	5.27, 8.31	< 0.001
Depression	no	5,020/15,930 (31.5%)	1.00		
	yes	19/57 (33.3%)	1.09	0.61, 1.86	0.768

Legend: CI – confidence interval; CLI – chronic limb-threatening ischaemia; COPD – chronic obstructive pulmonary disease; OR – odds ratio

#### 3.3.2 Results of univariate analysis of severe PAD

The results of univariate analysis showed that severe stages of PAD were associated with sex, age and the majority of comorbidities, except for coronary artery disease, cerebrovascular disease, COPD/asthma and depression (Table 7).

## 4 DISCUSSION

The main study results showed that hospitalisation rates were broadly stable between 2015 and 2022, with a transient reduction in 2020 and 2021 (the COVID-19 period). Hospitalised patients were predominantly men, and chronic limb-threatening ischaemia was associated with older age and a higher burden of recorded comorbidities than intermittent claudication.

As for hospitalisation rates, they reflect a substantial burden of PAD in Slovenia. While direct comparisons with other countries should be interpreted with caution (due to different methodologies and diverse outcome measures), the estimated hospitalisation rates for Slovenia (Table 3) are comparable to those from other European countries, such as France (128.7 per 100,000/year) (12) and Germany (204.4–231.7 per 100,000/year) (13), but are lower than in the United States (529–977 per 100,000/year) (14) — the latter likely reflecting an overuse of peripheral procedures in the fee-for-service healthcare environment of the United States (15). Hospitalisation rates in Slovenia increased from 2015 to 2020, mirroring a trend in increasing PAD hospitalisation rates in other countries (12,13,14) and reflecting the growing epidemiological burden of PAD (6). A meta-analysis of community-based studies between 2000 and 2010 revealed a 13–29% increase in PAD incidence (7) following coronary artery disease and stroke. This study provides the first comparison of the prevalence of peripheral artery disease between high-income countries (HIC, while the Global Burden of Disease, Injuries, and Risk Factors Study (5) estimated that the total number of patients with PAD almost doubled between 1990 and 2019 (from 65.8 to 113 million). The increasing epidemiological burden of PAD likely derives from ageing populations, as age-standardised prevalence rates decreased by 21.7% in the same period (5). This decrease, which may indicate better secular trends in PAD prevention and management, aligns with relatively stable age-standardised hospitalisation rates in our analysis. Conversely, there was a meaningful transient reduction in hospitalisation rates during the COVID-19 pandemic in 2020–2021. Similar observations — attributed to disruptions in hospital healthcare provision due to the pandemic — have been reported for several cardiovascular conditions (16), including PAD (17). In a large multi-centre cross-sectional analysis of hospital admissions for PAD in China, suspending non-urgent elective cases at medical institutions led to a 25% drop in PAD hospitalisations in the first three months of the pandemic outbreak, which was followed by a 10% uptick in the following months (17).

Results related to episodes with procedures showed that the vast majority of our PAD inpatient population underwent revascularisation, suggesting that invasive management is a major driver of inpatient care. Endovascular procedures accounted for the largest share across the study period, while the relative contributions of endovascular and open surgical approaches varied between years.

Analysis of adverse outcomes revealed that major amputation and in-hospital fatality were lower in 2022 than in 2015. However, both outcomes fluctuated over time and should be interpreted as descriptive signals that may reflect changes in case mix, access to care and pandemic-related disruptions. Overall, the observed amputation and in-hospital fatality were broadly comparable to estimates reported in other inpatient PAD studies (12,13,14).

As for costs, median reimbursement costs were 2.4- and 5.5-times lower than in Germany and the United States, respectively (adjusted for inflation and purchasing power parity) (13, 14, 18).

At the individual level, our study confirmed a strong male preponderance and high burden of comorbidities in patients hospitalised with PAD. In terms of sex differences, our analysis echoes previous studies of PAD inpatients (12,13,14). While the overall prevalence of PAD is similar in men and women at any given age (6), PAD in men has been associated with higher disease severity and disability (6), thus likely contributing to higher hospitalisations in men (5, 19). The latter may explain consistently higher proportions of men in analyses of hospitalised PAD patients (12,13,14), including ours.

In terms of comorbidities, patients with PAD have a higher frequency of diabetes mellitus, arterial hypertension, dyslipidemia, chronic kidney disease, and non-peripheral atherosclerosis—all conditions that are established risk factors for PAD onset and progression (6). In particular, diabetes mellitus and arterial hypertension (along with smoking) are major determinants of PAD, especially when compared to other forms of atherosclerotic vascular disease (20). However, the frequency of some chronic conditions in our dataset likely reflects under-recording in discharge coding. For context, population-level data from Slovenia suggest that hypertension affects roughly one-third of adults (21) and elevated total cholesterol is common (22), while diabetes frequency is estimated at around 7–9% in adults (23). Compared with these benchmarks, the recorded frequency of dyslipidemia and polyvascular disease in our study group appears low, supporting the interpretation that comorbidities captured solely from discharge diagnosis codes underestimate the true risk profile. The strong association between diabetes mellitus and PAD also represents a major target for population-level initiatives, such as the European Joint Action on CARdiovascular diseases and DIabetes (JACARDI) (24).

The findings of univariate analysis of risk factors associated with unfavourable outcome suggest that such outcomes in hospitalised PAD patients are closely related to older age, higher comorbidity burden and systemic cardiovascular risk profile. Because this analysis was univariate, these associations should be interpreted as exploratory and not considered independent predictors of an unfavourable outcome. Overall, patients hospitalised for PAD represent a patient population with a high burden of comorbidities, resource-intensive procedures, and unfavourable outcomes. The burden of inpatient PAD in Slovenia is substantial and comparable to estimates from other European countries, suggesting the need for strengthening integrated care through prevention, early detection and timely management of atherosclerotic vascular disease across all levels of care (9).

Our study has some potential limitations. First, one could argue that administrative data were used rather than a specific epidemiological study, e.g., a cohort study. However, the opportunistic and resource-intensive nature of cohort studies represents a major drawback for the timely appreciation of the current epidemiological burden of PAD, whereas routinely collected administrative data—albeit limited in scope (usually capturing more severe and symptomatic cases)—may provide timely estimates of the current PAD burden. Second, our scope was restricted to inpatient PAD episodes and therefore does not capture outpatient care, asymptomatic disease, or undiagnosed PAD. However, the purpose of the study was to provide a first estimation of the problem at the level of Slovenia as a whole, which should inform and guide further data collection/linking related to PAD. Third, diagnoses and comorbidities were derived from discharge coding submitted for reimbursement, which may be incomplete and can lead to misclassification or under-ascertainment of chronic conditions and non-peripheral atherosclerosis. However, our analysis was primarily focused on index diagnosis of PAD; while coding may underestimate the true prevalence of concomitant diagnoses in patients with PAD, it still provides an estimate of the comorbidity burden in PAD, particularly in CLI patients. Fourth, key determinants of PAD, such as smoking status, physical activity and diet were not available, and medication use could complement our data on comorbidities. At this stage, our study sought to provide a rough estimate of PAD in Slovenia; this limitation should nonetheless guide future analyses. Fifth, hospitalisations for non-insured patients (e.g., non-residents or tourists) could not be captured (10). However, given the very small proportion of non-insured residents in Slovenia, we believe that this limitation did not affect the results of our study. Sixth, we only used elementary analytical methods. Nonetheless, our study provides a first appraisal of the data availability and use, and more sophisticated methods were out of scope at this stage. Finally, our time series is very short, covering only an 8-year period. Nevertheless, our data provide important information as a basis for further research and perhaps for redesigning routine health data collection in the country. Despite the limitations, this nationwide study provides a timely foundation for clinical service planning and for targeted national-level interventions to reduce PAD burden.

Regarding clinical and public health implications, our findings highlight that PAD hospital care in Slovenia is resource-intensive and largely driven by revascularisation procedures. The substantial inpatient cost burden and the high proportions of patients with diabetes, hypertension and chronic kidney disease support the need for integrated vascular care pathways that emphasise secondary prevention, optimisation of cardiometabolic risk factors, and timely referral for limb-salvage evaluation. From a public health perspective, these data can inform prioritisation of prevention programmes targeting smoking, diabetes, and hypertension, and support planning for multidisciplinary diabetic-foot and limb-preservation services.

There are many opportunities to continue researching the burden of PAD in the future on both levels. At the population level, for example, it would be sensible to conduct a time-trend study by extending the time series; at the individual level, a multivariate association analysis in which comorbidities are considered confounding factors would be more than welcome. Future research should also extend the inpatient estimates by linking hospital discharge data with outpatient/primary care data, the national prescription (e-prescription) database and mortality registries to capture medication use, longitudinal outcomes after discharge and the full healthcare cost burden, as well as by linking with nationwide health behaviour surveys (25). Such linkage would also enable inclusion of key risk factors not available in discharge abstracts (e.g., smoking status) and facilitate validation studies of coding for PAD phenotypes and procedures against clinical records. Finally, further studies should assess regional variation, outpatient disease burden, medication use, and post-discharge outcomes by linking with complementary data sources.

## 5 CONCLUSION

Our nationwide analysis of an administrative hospital discharge database provides insight into the burden, direct inpatient reimbursement costs, comorbidity patterns and in-hospital outcomes of PAD hospitalisations in Slovenia. Hospitalised patients were predominantly men, and chronic limb-threatening ischaemia was associated with older age and a higher burden of recorded comorbidities than intermittent claudication. Hospitalisation rates were broadly stable between 2015 and 2022, with a transient reduction during the COVID-19 period. Major amputations and deaths were lower in 2022 than in 2015, but these outcomes fluctuated between years and should be interpreted as descriptive trends.
